# Raynaud’s Phenomenon and Limb Hypertrophy Presenting Phacomatosis Pigmentovascularis: A Rare Association

**DOI:** 10.7759/cureus.25900

**Published:** 2022-06-13

**Authors:** Dharmendra K Pipal, Rajendra K Pipal, Vijay Verma, Vibha Rani Pipal, Seema Yadav

**Affiliations:** 1 General, Colorectal and Minimal Access Surgery, All India Institute of Medical Sciences, Gorakhpur, Gorakhpur, IND; 2 Orthopedics, Geetanjali Medical College, Udaipur, IND; 3 General Surgery, Sarojini Naidu (SN) Medical College, Jodhpur, IND; 4 Obstetrics and Gynecology, All India Institute of Medical Sciences, Gorakhpur, Gorakhpur, IND; 5 Anesthesia and Critical Care, Jaipur National University (JNU) Hospital, Jaipur, IND

**Keywords:** phacomatosis pigmentovascularis, developmental malformation, capillary nevus, limb hypertrophy, raynaud's phenomena

## Abstract

Phacomatosis pigmentovascularis is a rare dermal disorder attributed to the presence of various nevi. These lesions exist since birth, so the patient remains well aware of them. Various systemic involvements may be associated with the nevus, but the association of Raynaud’s phenomenon is seldom reported. Our patient came with similar features and, on workup, no neurovascular compression was present, such as cervical rib or thoracic outlet syndrome. Therefore, he was managed conservatively and experienced improvement following the treatment. The objective of reporting this case is to highlight the association of Raynaud's phenomenon with such nervous lesions.

## Introduction

Phacomatosis pigmentovascularis (PPV), a rare sporadic disorder, is marked by different pigmented nevi in conjunction with a capillary nevus [1,2]. The disorder was first reported way back in 1920, and a detailed description was made in 1947 by Ota et al. Additionally, the disease is so rare that only nearly 200 cases have been reported so far globally [3,4]. Historically, the disease was classified using letters and numbers that were very hard to memorize. Happle, in 2005, reassessed various case reports published on PPV in the past and classified the PPV in a comprehensive and easy-to-remember manner [5,6]. This is a case of PPV in a young male with Raynaud's phenomenon and thickening of the limbs.

## Case presentation

**Figure 1:**
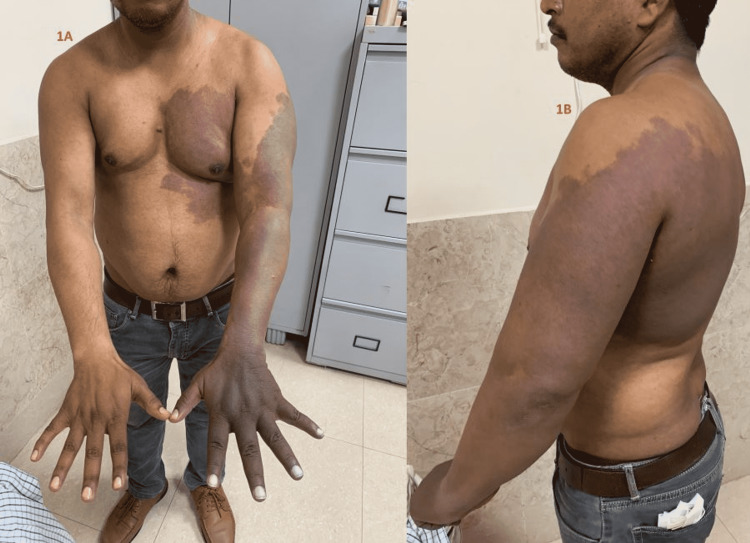
Extensive nevus located over the pectoral region, upper abdominal skin, almost covering the whole upper limb along with hypertrophy of biceps and rest of the left limb (A) and left side of the back extending to the upper trunk (B)

A 35-year- old male, resident of the northern part of India and a physical teacher by occupation presented to the surgical outpatient department with complaints of Raynaud-like symptoms in his left hand such as pain, tingling, and discoloration and swelling on exposure to cold. He used to travel on the air-conditioned bus for a few months for his job and noticed a slight pain and tingling during travel, which disappeared after getting off the bus indicating that the symptoms relieve in a warm environment. On examination, extensive bluish-black macular dermal pigmentation was present over the left pectoral region, upper abdominal skin, nearly whole upper limb, and the left side of the back extending up to the upper trunk along with limb hypertrophy measured by the increased girth of biceps and overall length of the left upper limb (Figures 1A, 1B).

**Figure 2:**
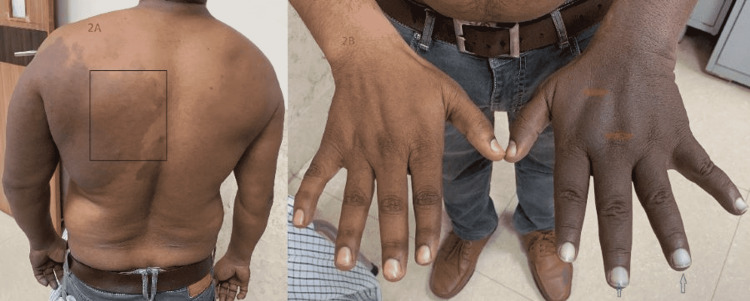
Red and pinkish lesions resembling the Port-wine stain on the left side of the posterior chest wall denoted by a black rectangle (A), and thick coarse skin and white pale nails denoted by red and green arrows, respectively (B)

On the left side of the posterior chest wall, medial to the extensive blue-black pigmentation, there were geographically shaped macular red and pinkish lesions resembling the port-wine stain (Figure 2A). All these lesions have been present since the birth of the patient. Additionally, the affected limb was pale at the time of presentation since the patient had been there for a couple of hours in our cold hospital environment compared to the normal limb. On detailed general examination, the hairs were normal, the nail bed was pale, and the left upper limb was a little more gigantic with thick coarse skin than the other limb (Figure 2B).

**Figure 3:**
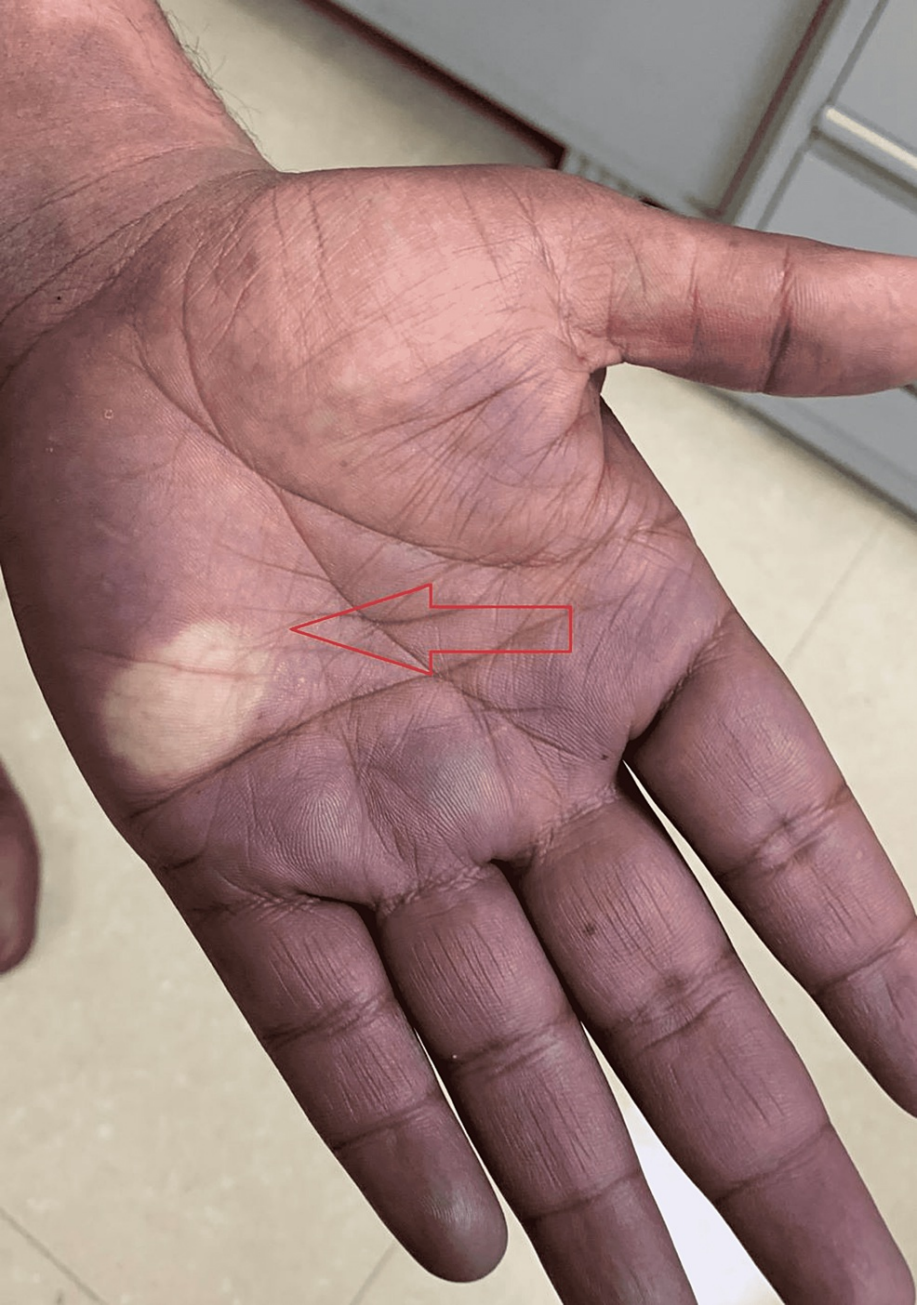
Blanching after applying pressure which is taking a long duration to turn pink; denoting Raynaud’s phenomenon

On keeping static pressure, blanching appeared, which took some time to turn pink over the palm and dorsum of the hand, suggesting Raynaud’s disease (Figure 3). Systemic examinations including cardiovascular, nervous and respiratory were normal. All joints including shoulder, elbow and wrist were also normal. An x-ray of the neck showed no cervical ribs. CT thorax for thoracic inlet and outlet and the CT angiography and antinuclear antibody (ANA) screening for connective tissue disorder was normal. He was also suggested for cervical sympathectomy but he refused. So, the patient was advised to avoid a cold environment and take cilostazol, pregabalin and pain killers. The patient showed improvement with the treatment.

## Discussion

Phacomatosis is a developmental anomaly/malformation of the ectoderm affecting various systems including the skin, eyes, brain, or the central nervous system (CNS) and very seldom affecting other viscera and bones. It is currently used to describe a few genetic diseases with multiple nevi and/or traces. In PPV, a vascular nevus coexists. As per the twin spotting theory, somatic replication exchanges autosomal recessive mutations on two adjacent foci that are responsible for the condition [7]. Cutaneous and systemic/syndromic variants are subtypes of phacomatosis. In 2005, Happle reclassified it into three types: phacomatosis cesioflammea, phacomatosis spilorosea, and phacomatosis cesiomarmorata. He also gave the terms, type I, and proposed type IV for the rare non-classifiable type. He also coined the phrase “phacomatosis multiplex” for situations that lacked a clear clinico-genetic entity. The commonest type is phacomatosis cesioflammea, which is distinguished by the presence of Mongolian spots and dermal melanocytosis, which are abnormal blue spots and port-wine stains [8]. “Caesius” is a Latin word that means bluish grey and is equivalent to the Mongolian term fuscoceruleus. A vascular nevus is a flammeus that is usually purple-red. Both skin lesions can develop alone or in conjunction with systemic involvement, such as CNS defects or ophthalmic anomalies, including melanosis bulbi or glaucoma [9,10], disproportionate limb length [11,12], dysplastic veinous or lymphatic system [13], or nevus anemicus [14,15]. Raynaud's phenomenon is a neurovascular symptom related to compression of subclavian vessels or brachial plexus in the thoracic outlet. Our patient did not have any such compression. So, whether there is an association between pigmented nevi and Raynaud's symptoms is a debatable issue as not much literature is available on it.

## Conclusions

Raynaud's phenomenon, asymmetrical limb hypertrophy, and thick and coarse skin were seen in this patient with PPV, which is extremely uncommon and has not been well-documented. The etiopathogenesis of Reynaud's phenomenon and melanocytic nevus needs to be better studied, especially when no apparent cause of neurovascular compression can be detected in a patient. Reporting this case to the literature may help in this field.
